# Purchasing reforms and tracking health resources, Kenya

**DOI:** 10.2471/BLT.19.239442

**Published:** 2019-12-12

**Authors:** Ileana Vilcu, Boniface Mbuthia, Nirmala Ravishankar

**Affiliations:** a ThinkWell, Rue du Mont-Blanc 15, 1201 Geneva, Switzerland.; b ThinkWell, Nairobi, Kenya.; c ThinkWell, Washington DC, United States of America.

## Abstract

As low- and middle-income countries undertake health financing reforms to achieve universal health coverage, there is renewed interest in making allocation of pooled funds to health-care providers more strategic. To make purchasing more strategic, countries are testing different provider payment methods. They therefore need comprehensive data on funding flows to health-care providers from different purchasers to inform decision on payment methods. Tracking funding flow is the focus of several health resource tracking tools including the System of Health Accounts and public expenditure tracking surveys. This study explores whether these health resource tracking tools generate the type of information needed to inform strategic purchasing reforms, using Kenya as an example. Our qualitative assessment of three counties in Kenya shows that different public purchasers, that is, county health departments and the national health insurance agency, pay public facilities through a variety of payment methods. Some of these flows are in-kind while others are financial transfers. The nature of flows and financial autonomy of facilities to retain and spend funds varies considerably across counties and levels of care. The government routinely undertakes different health resource tracking activities to inform health policy and planning. However, a good source for comprehensive data on the flow of funds to public facilities is still lacking, because these activities were not originally designed to offer such insights. We therefore argue that the methods could be enhanced to track such information and hence improve strategic purchasing. We also offer suggestions how this enhancement can be achieved.

## Résumé

Comme les pays à faible et moyen revenu se lancent dans des réformes de financement des soins de santé afin d’offrir une couverture maladie universelle, on constate un regain d’intérêt pour une répartition plus stratégique des fonds communs aux prestataires de soins de santé. Ces pays testent différentes méthodes de paiement des prestataires dans le but d’améliorer la stratégie d’achat. Ils ont donc besoin de données exhaustives sur les flux de financement entre ces prestataires de soins de santé et divers acquéreurs s’ils souhaitent prendre des décisions avisées dans ce domaine. La traçabilité du flux de financement est au cœur de plusieurs outils de suivi des ressources de santé, dont le Système des comptes de la santé et les enquêtes de suivi des dépenses publiques. Cette étude vise à déterminer si ces outils de suivi des ressources de santé génèrent le type d’information requis pour élaborer des stratégies d’achat adéquates, avec le Kenya en guise d’exemple. Notre analyse qualitative de trois comtés kényans démontre que différents acquéreurs publics, à savoir les autorités sanitaires des comtés et l’Agence nationale de l’assurance maladie, rémunèrent les établissements publics par le biais d’une série de méthodes de paiement. Certains de ces flux sont réglés en nature, tandis que d’autres sont des transferts financiers. La catégorie à laquelle appartiennent les flux ainsi que l’autonomie financière des établissements en termes de conservation et de dépense des fonds varient considérablement selon les comtés et les niveaux de soins. Le gouvernement mène systématiquement diverses activités de suivi des ressources de santé afin de mieux orienter la planification et la politique en la matière. Néanmoins, il manque toujours une source fiable de données exhaustives concernant le flux de financement des établissements publics car à l’origine, ces activités ne sont pas conçues pour livrer de telles observations. Nous affirmons dès lors que ces méthodes pourraient être optimisées pour récupérer ces informations et améliorer ainsi la stratégie d’achat. Nous formulons également des suggestions permettant de procéder à cette optimisation.

## Resumen

A medida que los países de ingresos bajos y medianos emprenden reformas de la financiación de la salud para lograr la cobertura sanitaria universal, se renueva el interés en que la asignación de fondos mancomunados a los proveedores de servicios de salud sea más estratégica. Para que las compras sean más estratégicas, los países están probando diferentes métodos de pago de los proveedores. Por lo tanto, necesitan datos completos sobre los flujos de financiación a los proveedores de servicios de salud de diferentes compradores para fundamentar la decisión sobre los métodos de pago. El seguimiento de los flujos de financiación es el objetivo de varias herramientas de seguimiento de los recursos sanitarios, incluidos el Sistema de Cuentas de Salud y las encuestas de seguimiento del gasto público. Este estudio explora si estas herramientas de seguimiento de recursos sanitarios generan el tipo de información necesaria para fundamentar las reformas de compras estratégicas, utilizando como ejemplo a Kenia. Nuestra evaluación cualitativa de tres condados de Kenia muestra que los diferentes compradores públicos, es decir, los departamentos de salud de los condados y la agencia nacional de seguro de salud, pagan a las instalaciones públicas a través de una variedad de métodos de pago. Algunos de estos flujos son en especie, mientras que otros son transferencias financieras. La naturaleza de los flujos y la autonomía financiera de los centros para retener y gastar los fondos varía considerablemente entre los condados y los niveles de atención. El gobierno lleva a cabo continuamente diferentes actividades de seguimiento de los recursos sanitarios para fundamentar las políticas y la planificación sanitaria. Sin embargo, sigue faltando una buena fuente de datos completos sobre el flujo de fondos a las instalaciones públicas, ya que estas actividades no se diseñaron originalmente para ofrecer este tipo de información. Por lo tanto, se argumenta que los métodos podrían mejorarse para hacer un seguimiento de dicha información y, en consecuencia, mejorar las compras estratégicas. También se ofrecen sugerencias sobre cómo se puede lograr esta mejora.

## ملخص

نظرًا لأن البلدان ذات الدخل المنخفض والدخل المتوسطة تضطلع بإصلاحات في التمويل الصحي لتحقيق تغطية صحية شاملة، فإن هناك اهتمام متجدد بجعل تخصيص الأموال المجمعة لمقدمي الرعاية الصحية أمراً أكثر استراتيجية. ولجعل الشراء أمراً أكثر استراتيجية، فإن البلدان تختبر يختبرون طرق دفع مختلفة لمقدمي الرعاية. وهم بالتالي يحتاجون إلى بيانات شاملة عن تدفقات التمويل إلى مقدمي الرعاية الصحية من مختلف المشترين، وذلك لاتخاذ قرار بشأن طرق الدفع عن دراية. تتبع تدفق التمويل هو محور العديد من أدوات تتبع الموارد الصحية التي تشمل نظام الحسابات الصحية ومسوحات تتبع الإنفاق العام. تستكشف هذه الدراسة ما إذا كانت أدوات تتبع الموارد الصحية تلك تولد نوع المعلومات اللازمة لتوعية إصلاحات الشراء الاستراتيجية، مع اتخاذ كينيا كمثال. يوضح تقييمنا النوعي لثلاث مقاطعات في كينيا أن المشترين العموميين المختلفين، أي إدارات الصحة بالمقاطعة ووكالة التأمين الصحي الوطنية، تدفع المرافق العامة من خلال مجموعة متنوعة من وسائل الدفع. بعض هذه التدفقات عينية بينما البعض الآخر عبارة عن تحويلات مالية. إن طبيعة التدفقات والاستقلالية المالية للمرافق للاحتفاظ بالأموال وإنفاقها، تتنوع بشكل كبير عبر المقاطعات ومستويات الرعاية. تضطلع الحكومة بشكل روتيني بأنشطة لتتبع الموارد الصحية المختلفة وذلك لتوعية السياسة الصحية والتخطيط الصحي. ومع ذلك، فإنه مازال هناك حاجة لمصدر جيد للبيانات الشاملة عن تدفق الأموال إلى المرافق العامة، لأن هذه الأنشطة لم تكن مصممة في الأساس لتقديم مثل هذه الأفكار. لذلك فإننا نزعم أن الأساليب يمكن تعزيزها لتتبع مثل هذه المعلومات، وبالتالي تحسين الشراء الاستراتيجي. كما نقدم اقتراحات حول كيفية تحقيق هذا التحسن.

## 摘要

随着中低收入国家为实现全民健康覆盖而进行医疗筹资改革，人们对将筹集到的资金分配给医疗护理提供者的战略意义重新产生兴趣。为了促使采购更具战略意义，各国正在检测不同提供者的支付方式。因此，各国需要不同购买者向护理提供者支付的资金流动的全面数据，以了解与支付方法决策有关的信息。追踪资金流是多种卫生资源追踪工具（包括卫生核算系统）和公共支出追踪调查的重点。本项研究以肯尼亚为例，探讨了这些卫生资源追踪工具是否生成了实现战略性采购改革所需的信息类型。根据我们在肯尼亚的三个地区开展的定性评估，不同的公共采购机构（即地区卫生部门和国家健康保险机构）采用多种不同的支付方式以支付公共设施费用。其中一些采用实物转移作为支付方式，而其他则采用支付资金的方式。在各个地区和医疗机构之间，保留和支出资金导致的设施的流动性质和财政自主权差异很大。政府定期开展各种卫生资源追踪活动，说明卫生政策和计划。但是，仍然缺少有关全面公共设施资金流相关数据的良好来源，因为设计这些活动的目的最初并非是为了提供此类见解信息。因此，我们认为可改进这些方法以追踪此类信息，从而提高战略性采购水平。我们还就如何完成此类改进提供了建议。

## Резюме

Проводимые реформы в области финансирования здравоохранения в странах со средним и низким уровнем дохода с целью достичь всеобщего охвата услугами здравоохранения способствуют выработке стратегии выделения средств поставщикам медицинских услуг из объединенных фондов. Чтобы разработать систему стратегического планирования закупок, страны испытывают различные методы оплаты услуг провайдеров. Поэтому странам необходима исчерпывающая информация о потоках финансирования, получаемых поставщиками медицинских услуг от разных потребителей, для принятия информированных решений о методах оплаты. Отслеживание потоков финансирования осуществляется с использованием нескольких инструментов отслеживания ресурсов в сфере здравоохранения, включая систему счетов здравоохранения и исследования структуры государственных расходов. Исследование рассматривает вопрос о том, предоставляют ли указанные инструменты отслеживания ресурсов здравоохранения информацию, необходимую для информационной поддержки реформ стратегического планирования закупок, на примере Кении. Проведенная авторами качественная оценка трех округов в Кении показывает, что разные государственные закупочные организации, а именно окружные отделы здравоохранения и национальное агентство медицинского страхования, оплачивают работу государственных организаций самыми разнообразными методами. Некоторые из денежных потоков имеют натуральное выражение, а другие поступают в результате перевода финансовых средств. Характер денежных потоков и уровень финансовой самостоятельности учреждений в части сохранения за собой этих средств и возможности их расходования сильно разнятся от округа к округу и зависят от уровня предоставляемых услуг. Правительство регулярно предпринимает меры по отслеживанию разнообразных ресурсов в сфере здравоохранения, чтобы получить информацию, необходимую для разработки политики и для планирования в этой области. Тем не менее надежный источник исчерпывающей информации о финансовых потоках, поступающих в государственные учреждения, по-прежнему отсутствует, так как подобные виды деятельности изначально не предусматривали получения сведений такого рода. В этой связи авторы считают возможным совершенствовать методы, что позволит отслеживать необходимую информацию и улучшить систему стратегического планирования закупок. Авторы также предлагают способы такого совершенствования.

## Introduction

As low- and middle-income countries undertake health financing reforms to achieve universal health coverage (UHC), there is renewed interest in making allocation of pooled funds to health-care providers more strategic.^1^^–^^3^ Such strategic purchasing is about health ministries, insurance agencies and other purchasers making key decisions about what services to buy, which providers to contract and how to pay providers based on information about provider performance and population health needs.^2^ Making these decisions demands a variety of data. This article focuses on data to inform decisions around payments to health-care providers.

Recent work on provider payment methods has shown that providers in most low- and middle-income countries receive a mix of payments from different purchasers under a variety of arrangements.^3^^,^^4^ To explore the extent to which the provider incentives created are aligned with desired outcomes, all flows of funding to a provider must be analysed.^1^ Since these tracking analyses need to be populated with information about all funding flows, the information required goes beyond what any individual purchaser possesses from claims data, budget documents or financial reports. Combining this available information from individual purchasers with data about provider behaviour and performance would allow purchasers to assess and improve provider payment methods.

Tracking the flow of funds is the focus of several health resource tracking frameworks and methods.^5^^,^^6^ Common methods for tracking the flow of resources, such as: the System of Health Accounts; Public Expenditure Tracking Surveys; Public Expenditure Reviews; Joint Reporting Form for immunization; and the National AIDS Spending Assessment, answer a range of important policy questions about health resource flows.^6^ Here we explore whether these methods provide comprehensive information about the flow of funds to health-care providers to inform the purchasing reforms that low- and middle-income countries are undertaking to achieve UHC.

We first describe what type of information is needed, and why, to be able to track financial flows to health-care providers. Then we discuss why the existing health resource tracking tools are not designed to produce the necessary information. We finally suggest how the existing tools could be improved to generate data to inform decisions about provider payments. To make this case, we describe a project we are currently working on, that is, to strengthen strategic health purchasing for primary health care in Kenya.

## The purchasing context

Historically, governments in most low- and middle-income countries established a national health service, wherein a government department, typically a health ministry at the national or sub-national level, allocated general revenue through line-item budgets to cover staff salaries, medicines and operating costs for a network of government-owned health facilities. Some governments also had parallel risk pooling arrangements, including social health insurance for formal sector workers, and many governments eventually introduced user fees in public facilities to mobilize more financing for the health sector.^7^

Countries are now undertaking reforms that move them from passively paying facilities based on pre-determined budgets or bills they present to the purchaser, to more strategic forms of purchasing. These forms of purchasing involve decision-making about the benefit package, providers contracted and payment methods.^1^^–^^3^ Many governments have initiated government-funded health insurance, wherein a public health insurance fund contracts and pays public and private providers to deliver a defined set of services to all citizens.^8^ Several countries, especially in Africa, have either capped or abolished user fees in the public sector, and introduced payments to public facilities to reimburse them for the fees forgone. Some governments have also implemented new performance-based financing programmes that introduce payments to facilities and/or providers that are more explicitly linked to outputs.^9^^–^^12^

In most countries, these reforms have resulted in complex flows of funds. Public sector facilities in many low- and middle-income countries continue to receive input-based budgetary allocations for salaries, drugs, and operating costs from the health ministry, as well as additional payments for delivering certain services from one or more purchasers. Another example of mixed provider payment is when multiple purchasers or the same purchaser implementing multiple schemes pay a provider different rates for the same service.^4^ Having a mix of funding flows raises two questions: are these different flows coherent; and are payments of some incentives difficult to follow if other larger incentives payments mask these payments. There are several guides and manuals for analysing provider payment methods, including a recent guide by the World Health Organization that offers an approach for analysing the full range of funding flows to providers.^4^ These guides and manuals largely employ mixed methods, drawing from available data sources to explore the flow of funds.

Another question is how good are the existing data sources to track the complex flow of funds to providers? Furthermore, for governments that are already undertaking different health resource tracking activities, do they obtain sufficient data of good quality to analyse multiple funding flows? The goal for collecting this type of data would be to provide insights into the flow of funds to providers of different types rather than for tracking flows to individual providers in the country.

## Resource tracking methods

The term health resource tracking covers a range of frameworks to collect, analyse and present information about health spending. Some frameworks account for all health spending, while others focus on specific diseases or health areas. Some frameworks focus on public spending, while others track public, private and donor financing. The System of Health Accounts and Public Expenditure Tracking Surveys are the methods most relevant for tracking the full range of flows to providers, and these methods are commonly used in low- and middle-income countries.^6^

The System of Health Accounts is a framework to measure total health spending in a country at one-time point, and disaggregate the spending along different dimensions, such as source of revenue, financing scheme, health function, type of providers and factors of provision.^13^ This framework uses a range of primary data sources including government budget documents, household surveys and claims data from insurers. The framework has been implemented by 148 countries.^6^ By offering standardized definitions and classifications, health accounts allow for comparison of health expenditure between countries and over time. While the framework has general guidelines on the core dimensions that countries should use, countries can choose which dimensions to use for disaggregating spending data as well as the level of detail to provide for each dimension.

While results of health accounts exercises are an invaluable source of information about health spending patterns in a country, the framework is not designed to provide a granular assessment of revenue and expenditure from a facility perspective because it aggregates spending patterns.^6^ For example, the results can show the total amount of spending at hospitals versus health centres, or how much the health ministry paid for health-care delivery at public hospitals as a whole. The framework is not, however, designed to provide insights into what revenue looks like for the average hospital or health centre, and therefore cannot provide information about the relative strength of different incentives created by payment methods from the perspective of the provider.

Another health resource tracking method, the Public Expenditure Tracking Surveys, is designed to track and quantify flow of funds from the national treasury through various government agencies to the final points of service delivery. Using the method typically entails data collection at the central, sub-national and facility levels, and the method is often implemented alongside the Service Delivery Indicator Survey.^6^ The method is a useful tool for assessing the links between public financial management and service delivery, and explaining planning and management capacities of various government entities, delays in disbursements and leakage of funds.^6^^,^^14^ Since the World Bank first applied the method in 1996, it has been implemented across a range of countries in Africa and Asia. The surveys should have five broad stages: defining objectives; mapping flows; measuring leakages; presenting findings; and informing policy.^15^ Beyond these broad stages, countries have the flexibility to modify the survey to reflect the local context and policy priorities. As a result, there is variability across countries’ results in terms of whether the survey provides a complete picture of facility financing or focuses only on specific types of funding flows.

## A Kenyan perspective

In the post-colonial period, the Kenyan government has financed health-care delivery through a traditional national health service. All services were free until 1988, when user fees were introduced. Public facilities could retain these funds as well as any reimbursement they received from the National Hospital Insurance Fund, Kenya’s social health insurance agency. They used the revenue to finance their operating costs, while the health ministry paid for health workers’ salaries and centrally procured drugs. While the government introduced waivers for various maternal and child health services during the 1990s, studies showed that overall use of health-care services went down because of the fees.^16^

In 2004, the government introduced the 10/20 policy, which replaced user fees at government-owned health centres and dispensaries with a single one-time registration fee of 20 shillings (0.2 United States dollars, US$) at the health centres and 10 shilling (US$ 0.01) at the dispensaries.^17^ To compensate for the loss in revenues from user fees, the government, with support from donors, set up the Health Sector Services Fund in 2009 to transfer resources directly to these facilities.^18^^,^^19^ In the same year, the government established the Hospital Management Support Fund to compensate hospitals.^20^ The 2012 public expenditure tracking survey found that only 112 (45%) of 249 surveyed dispensaries and health centres complied with the 10/20 policy. The weighted results showed that user fees accounted for 53% of the operating budget of these facilities, while the Health Sector Services Fund accounted for 31% of the health centres’ budgets and 40% of the dispensaries’ budgets. User fees accounted for 70% of the revenue of public hospitals, while the Hospital Management Support Fund and the National Hospital Insurance Fund payments accounted for only 14% and 5%, respectively.^21^

In 2013, Kenya transitioned to a devolved system of government. Under the new arrangements, county governments control all primary and secondary health service delivery. Through line-item budgets, they pay for services that are provided by a network of public facilities. As per the constitution, all funds collected by public facilities are to be remitted to the county government unless the county passes legislation allowing facilities to retain own-source revenue to offset their costs. In 2013, the national government also abolished all user fees at health centres and dispensaries in the public sector, as well as user fees for maternal health services at public hospitals. Instead of compensating facilities for the loss of revenue from user fees, as it had done under the mechanisms of the Health Sector Services Fund and the Hospital Management Support Fund, the national government now started giving the funds to the county governments in the form of intergovernmental transfers. In 2017, the health ministry transferred the free maternity scheme to the National Hospital Insurance Fund, at which point it was renamed Linda Mama. The National Hospital Insurance Fund now directly contracts and pays both public and private facilities for maternal health services.^18^^,^^22^

**Fig. 1:**
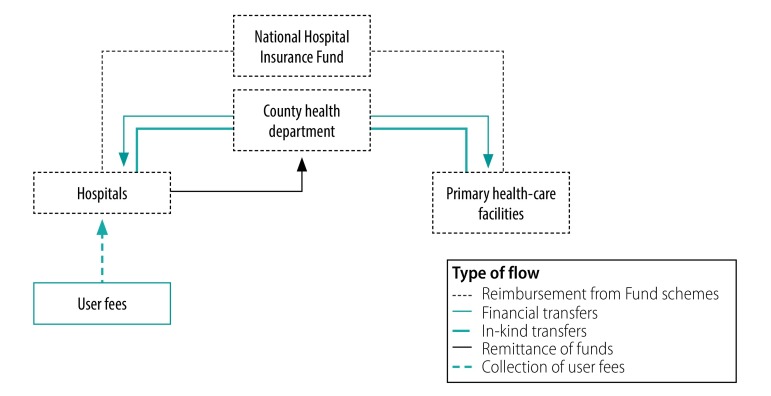
Flow of funds in Isiolo, Kilifi and Makueni counties, Kenya, 2019

Notes: Hospitals in Kilifi and Isiolo, but not Makueni, are required to remit all the funds they collect to the county treasury. Only Makueni is making financial transfers from the county health department to the hospitals. An example of a financial transfer is a payment for providing services under an insurance scheme designed and implemented by the county health department. Examples of in-kind transfers are salary payments and drug procurement.

Against this background, the project team did a rapid landscaping study in our three project counties (Isiolo, Kilifi and Makueni) between November 2018 and March 2019, to increase the understanding of purchasing at the county level. The exercise yielded several interesting insights. First, the three counties vary considerably in how they handle facility revenues from various sources (Fig. 1). Through an executive order by the county government, Makueni has allowed all public health facilities to retain and spend any funds they collect. Kilifi has enacted a legislation creating a fund where all user fees from hospitals would be remitted and subsequently used to finance facility improvement plans, but the fund has not been established to date. Isiolo has made no provision for facilities to retain funds. Hence, hospitals in both Kilifi and Isiolo are required to remit all the funds they collect from user fees and the National Hospital Insurance Fund payments to the county treasury, and the county government pays directly for any expenses for these health facilities.^23^^,^^24^ They have completely lost the financial autonomy they had before devolution. In contrast, hospitals in Makueni control their own budget, which is financed through the user fees and claims reimbursements they collect.

Second, in all three counties, the county governments allow public health centres and dispensaries to retain all funds they collect from the National Hospital Insurance Fund. The county governments also give these facilities funds drawn from specific grants that the county governments receive from the national government. Primary care facilities spend funds according to the investments plans they develop, but only after they receive approval to incur expenditure from the county health department, which happens on a quarterly basis.

Third, primary health facilities in all three counties indicated that reimbursements from the National Hospital Insurance Fund are less than expected and subject to huge delays. Thus, these revenues are not perceived as a reliable source of funding. Public health facilities do not follow-up with the National Hospital Insurance Fund to reconcile claims and reimbursements on a regular basis in Isiolo and Kilifi. Therefore, health facilities are losing funds because they continue to provide service under the *Linda Mama* free maternity scheme without receiving reimbursements. Therefore, measures to strengthen accountability so health facilities at all levels are reimbursed for the services they provide are needed. The National Hospital Insurance Fund reimbursement rates for maternity services under the *Linda Mama* scheme is lower than the rates the National Hospital Insurance Fund offers for the same services under other insurance schemes, which distorts the incentives providers must cater to *Linda Mama* beneficiaries.^25^

While the study allowed us to describe the flow of funds in each of the three counties, we struggled to quantify or track the full range of funding flows to providers due to the paucity of data. Kenya has implemented five health accounts exercises.^26^^,^^27^ The most recent rounds provide aggregates for core health accounts dimensions, specifically source of revenue, financing scheme, provider, and function, but do not provide any cross tabulations. The last round in 2015/2016 included county health accounts, but these again only offer aggregate spending information and do not show how much different providers received from different schemes.

While county budget documents provide aggregate allocations for salaries, commodities and facility maintenance costs, these are not disaggregated by facilities. Facility budgets are not a reliable source of information about the flow of funds to facility for several reasons. First, hospitals in both counties Isiolo and Kilifi remit all own-source revenue to the county, which incurs expenses on their behalf. Hence, the budgets hospitals prepare and submit to the counties are wish lists and do not track facility revenue and expenditure in a systematic manner. Primary health-care facilities, which retain and spend funds in all three counties, prepare operating budgets, but these do not reflect any of their salary or commodity costs.

The latest Public Expenditure Tracking Surveys, conducted in 2012, offered detailed information about how much revenue facilities generated from different sources like user fees and the National Hospital Insurance Fund reimbursements, but did not consider other in-kind flows to the facility, such as health workers’ salaries and drugs paid by the health ministry’s budget or donor support. The health ministry is planning health resource tracking exercises in 2020 with support from the World Health Organization and other partners, using the System of Health Accounts framework and the Public Expenditure Tracking Surveys. These exercises represent a promising opportunity to not only collect granular information about the full range of resource flows to providers, but also link to health resource tracking exercises to provide a comprehensive view of health financing in the country.

## A call for resource tracking

Our landscaping exercise to explore county purchasing practices in Kenya revealed an information gap on the flow of funds to providers. In the absence of regularly conducted studies that triangulate between different information sources like claims data from the National Hospital Insurance Fund, as well as county and facility budget documents, and financial accounting systems, a complete mapping of financial flows at the facility-level proved to be very difficult. While our study and others^25^ have collected qualitative information describing the flows, the relative size of any given flow to the facility is not discernible. Understanding how much facilities receive from different sources of revenue, insurance claims, financial and in-kind transfers from the county government, and user fees, as well as how these funds reach the facility, how much autonomy the facilities have to spend the funds, and types of costs they can incur is critical for ongoing policy discussions on UHC reforms in the country.

Many low- and middle-income countries implement the System of Health Accounts, Public Expenditure Tracking Surveys and other health resource tracking activities to analyse health spending. Expanding one or more of these existing platforms to track the flow of funds to providers, rather than introducing a new method, seems desirable from the perspective of both efficiency and capacity. We urge the team that will undertake the upcoming health recourse tracking activities and international agencies supporting them to use the opportunity to explore how the two methods could be enhanced to track the funding flows to health-care providers in a more comprehensive manner. The System of Health Accounts method allows for presentation of data from various perspectives using cross-tabulations between different dimensions; this is one promising way in which the Kenyan government could get more information from health accounts. Second, past Public Expenditure Tracking Surveys have focused on a subset of financial flows and not attempted to measure in-kind transfers to health facilities. We recommend that the upcoming Public Expenditure Tracking Surveys aim to account for all financial and in-kind transfers to facilities from different purchasers. These extensions would allow the next health resource tracking activities to generate valuable information about the flow of funds to providers to guide ongoing discussions about strategic purchasing reforms in the country, and in turn benefit other countries in the African region and beyond.
